# Breastfeeding competency and its influencing factors among pregnant women in third trimester pregnancy: a cross-sectional study

**DOI:** 10.1038/s41598-023-28477-4

**Published:** 2023-01-23

**Authors:** Yu Wu, Wenwen Liu, Xia Liu, Yunfeng Li, Ying Wang, Yanxin Chu, Qian Pi, Xin Zhao, Jinxiang Lu, Aihua Wang

**Affiliations:** 1 Delivery Room, Shandong Provincial Hospital Affiliated to Shandong First Medical University, 9677 Jingshi Street, Jinan, 250021 Shandong China; 2 School of Nursing, Weifang Medical University, 7166 Baotong West Street, Weifang, 261053 Shandong China; 3 Department of Nursing, Shandong Provincial Hospital Affiliated to Shandong First Medical University, 9677 Jingshi Street, Jinan, 250021 Shandong China; 4 Shandong Provincial Qianfoshan Hospital, The First Hospital Affiliated with Shandong First Medical University, 16766 Jingshi Street, Jinan, 250014 Shandong China; 5 People’s Hospital of Lixia District of Jinan, 73 Wenhua West Street, Jinan, 250000 Shandong China

**Keywords:** Paediatric research, Nutrition, Risk factors

## Abstract

Competency is closely related to the occurrence of the behavior. Breastfeeding competence is the mastery of different breastfeeding factors which intervene in breastfeeding behavior. Breastfeeding competence could improve the breastfeeding behavior. However, few studies have paid attention to the status and the influencing factors of breastfeeding competency. The breastfeeding competency of pregnant women in third trimester pregnancy has the greatest impact on breastfeeding behavior after childbirth. Therefore, the objective of this study were to investigate the breastfeeding competency level and independent risk factors for breastfeeding competency among pregnant women in third trimester pregnancy. A cross-sectional survey method and convenience sampling method was used in the study. The general information questionnaire including age, gestational week, educational background, and so on were used to investigate the general information of pregnant women and their husbands. A breastfeeding competency scale (BCS) was used to investigate the breastfeeding competency of pregnant women. The total score of the BCS ranges from 38 to 190, with higher scores indicating greater breastfeeding competency. Lower level, medium level and higher level are 38–89, 90–140 and 141–190 respectively. Type-D Scale-14 (DS14) was used to investigate the type D personality of pregnant women. A multivariable linear regression was used to examine the independent predictors of breastfeeding competency. A total of 550 questionnaires were collected and finally 525 effective questionnaires were collected. The age of 525 pregnant women is (30.24 ± 3.954) years old. The breastfeeding competency score of pregnant women was (134 ± 19.741). Multivariable linear regression analysis showed that higher breastfeeding competency in pregnant women were reported among pregnant women who gestational age ≥ 256 days (37 weeks) (B = 8.494, p < 0.001), the previous breastfeeding experience were exclusive breastfeeding (B = 17.384, p < 0.001) and partial breastfeeding (B = 16.878, p < 0.001), participating in pregnant women school 2–3 times (B = 10.968, p = 0.013) and ≥ 5 times (B = 13.731, p = 0.034). Pregnant women with lower breastfeeding competency were found in women who were judged to have type D personality (B = − 6.358, p < 0.001). The result can explain 25.8% of the variation in the total breastfeeding competency score. This should be considered an important issue by maternal and child health care in the medical system that the moderate level of breastfeeding capacity among pregnant women. Differentiated and targeted breastfeeding support and services for pregnant women should be carried out based on influencing factors of breastfeeding competency.

## Introduction

Breast milk is a normal special food for infants which provides all the nutrients for the first 6 months of life^1,2^. Breast milk is the best nutrition for infants which can improve health across the life span and bring enormous benefits to society and families^3,4^. Promoting breastfeeding can increase family income by protecting the growth and development of infants and promoting women's postpartum recovery, which can bring more labor and demographic dividends to society^5–7^. It can also achieve the purpose of reducing family and social medical expenses by decreasing the risk of short-term and long-term diseases of women and infants^8,9^.

Despite the many benefits of breastfeeding, breastfeeding practices in most countries are still below the recommended standards by the World Health Organization^10^. Globally, approximately 30% countries with an exclusive breastfeeding rate of less than 20% from 0 to 5 months, of which low and middle-income countries breastfeed longer than high-income countries^11,12^. 25.3% infants are exclusive breastfed at 0 to 5 months in China, and the prevalence of breastfeeding at 6 months is 34.7% in USA. In Turkey, the rate of infants who are exclusive breastfed is 46.90%^12^. It brings a hidden danger to maternal and infant health, family security, and social development. The issues among physiology, psychology, and social cultures such as mother-infant separation, low breastfeeding knowledge, and self-efficacy could be experienced by pregnant women^13^.

Almost all pregnant women have enough milk for breastfeeding, and only 5% of pregnant women may experience physiological insufficient milk^14^. But breastfeeding, as a complex behavior of women, is affected by many factors such as physiology, psychology, and social culture. Predicting breastfeeding behavior solely from breastfeeding knowledge, skills, intentions or social culture is not comprehensive^9^. Conceptual Model of Components of an Enabling Environment for Breastfeeding was established by Nigel C Rollins to explain factors that influence early initiation to continue breastfeeding^9^. Structural level, settings factor, and individual level were included in this conceptual Model. The structural level includes the attitude towards breastfeeding in the sociocultural and market context^15^. Settings factor include legislation, policy, support from health systems, and workplace protections for breastfeeding practices. The individual level includes physiology, psychology, breastfeeding skills, lifestyle of pregnant women, etc.^16^. Therefore, predicting the occurrence of breastfeeding behaviors and positive breastfeeding experiences requires a comprehensive evaluation of psychological, physical, and social background.

Based on the perspective of competence, breastfeeding competence is defined as the mastery of different breastfeeding factors, such as cognition, knowledge, and skills, which intervene in breastfeeding behavior on the basis of understanding, controlling, and grasping these factors at the same time^17^. Research from the point of breastfeeding competency and unified control of various factors could maximize the effect of breastfeeding intervention and achieve the purpose of effectively promoting breastfeeding^17,18^. However, most studies focus on breastfeeding outcomes and their influencing factors^19,20^, few studies focus on breastfeeding based on the perspective of competence and analyze the influencing factors of breastfeeding competency at present. Therefore, the objective of the present study was to identifying the status and independent risk factors for breastfeeding competency among pregnant women in third trimester pregnancy, to provide references for the development and update of breastfeeding competency and behavior management in the future.

## Methods

### Setting, participants and data collection

This cross-sectional survey was carried out from September 2019 to November 2019 in Weifang, China. Pregnant women who recruited from two provincial general hospitals by convenient sampling were invited to participate in the study. The questionnaires were collected by a combination of online questionnaires and paper questionnaires, and participants chose either one method to complete in the department of gynaecology and obstetrics. The inclusion criteria which the participants should meet were whole-pregnancy examinations performed in this hospital, being in the third trimester, and volunteering to participate independently. The exclusion criteria were inability to understand the content of the scale after detailed explanation and/or contraindications to breastfeeding.

In the department of gynaecology and obstetrics, consent forms were distributed to pregnant women who met the inclusion and exclusion criteria, while standardized explanations of the research objectives, procedures, and questionnaires were given to the pregnant women to ensure their full understanding. After agreeing to participate, pregnant women completed questionnaires using online questionnaires and paper questionnaires. The questionnaires were completed on the day or they were sent to the researchers during the next hospital visit. The researchers collected the online and paper questionnaires on the day or next hospital visit, strictly reviewed the filling of the questionnaires, and summarized the data of online and paper questionnaires. We increased the study size to 550 to allow for possible data loss due to clinical conditions. Finally, 525 effective questionnaires were collected. The response rate was 95.45%.

### Measures

#### General information

General information of pregnant women consisted of age (< 35, ≥ 35), gestational week (days, < 259, ≥ 259), educational background (College diploma below, College diploma, Bachelor, Master or higher), occupation (Administrative, medical, Clerk, Professional, Freelance and others), monthly income (yuan, < 5000, 5000–, 10,000–, 15,000–,  ≥ 20,000), Husband’s educational background (College diploma below, College diploma, Bachelor, Master or higher), husband’s occupation (Administrative, medical, Clerk, Professional, Freelance and others), previous breastfeeding experience (none, first born, exclusive breastfeeding, partial breastfeeding, artificial feeding), Residency (registered urban residents, registered rural residence), Pregnant women school attendance (times, 0, 1–2, 3–4,  ≥ 5), Application (APP) usage (days per week, 0, 1–2, 3–4, ≥ 5).

#### Breastfeeding competency scale (BCS)

The 38-item Breastfeeding Competency Scale was developed and based on the competency iceberg model and referenced the other scale^17,21–23^. The competency iceberg model was established to explain factors that development and translation of competency, which includes four part: knowledge, skills, self-concept, traits,and motivation^17,24^. BCS could break down breastfeeding competency into different parts that are easier to assess and reliably represent and evaluate breastfeeding competency^17^. Each item is rated on a five-point Likert scale ranging from 1 ‘completely no corresponding’ to 5 ‘completely corresponding’. The total score of the BCS ranges from 38 to 190, with higher scores indicating greater breastfeeding competency. Using the fractional three-point method, 38–89 as a lower level, 90–140 as a medium level, and 141–190 as a higher level. The The BCS has good reliability and validity (Cronbach’ s alpha of 0.970 and CFA model showed that the 4-factor model fits the data well). The Cronbach’ s alpha coefficients of BCS is 0.925 in this study.

#### Type-D Scale-14 (DS14)

Type D personality was measured with Type-D Scale-14 (DS14), including both subscales of negative emotion and social inhibition. Participants were asked to rate the truthfulness of statements from 0 to 4. Type D personality was diagnosed when the negative affect scale and the social inhibition scale have scores of 10 or more. Cronbach's alpha coefficient of the scale was 0.87, indicating that it has good reliability^25^.

### Ethical considerations

The protocol for this study was approved by the Medical Ethics Committee of Weifang Medical University (2019SL060). Written informed consents were obtained from the all participants and their personal information was kept strictly confidential. All methods were performed in accordance with the relevant guidelines and regulations including the Declaration of Helsinki.

### Statistical analysis

The statistical analysis was using SPSS version 23.0 or AMOS version 23.0. Descriptive analyses were conducted to present the frequency (percentage) and means (standard deviations, SDs) of the general information about pregnant women. Univariate analysis of breastfeeding competency level was analyzed using t-test and analysis of variance (ANOVA). Analysis of multicollinearity among different factors using tolerance and VIF value. A multivariable linear regression with the enter method was used to examine the independent predictors of breastfeeding competency. P < 0.05 was chosen for the statistical significance assessment.

## Results

### Characteristics of participants

The age of 525 pregnant women is (30.24 ± 3.954) years old and the gestational week is (243.03 ± 22.556) days, of which 291 (55.43%) people with registered urban residents and 234 (44.57%) people with registered rural residence. There were 66 (12.57%) participants with college diploma below, 143 (27.24%) participants with college diploma, 237 (45.41%) participants with bachelor, and 70 (13.33%) participants with master or higher. The husband’ s educational background is a college diploma below 68 (12.95%), college diploma 142 (27.05%), bachelor 233 (44.38%), and master or higher 82 (15.62%). A total of 183 (34.56%) people were type D personality, and 342 (65.14%) people were non-type D personality (See Table 1).

**Table 1:** Univariate analysis of breastfeeding competency level (n = 543).

Characteristics	Number	Percent (%)	Score	t/r	P
Age					
< 35	449	85.52	137.70 ± 21.176	-3.912	< 0.001
≥ 35	76	14.48	146.184 ± 16.775		
Gestational week (days)					
< 259 (36^+6^)	366	69.71	136.25 ± 20.865	-4.565	< 0.001
≥ 259 (37)	159	30.29	145.10 ± 19.337		
Educational background					
College diploma below	66	12.57	143.70 ± 19.914	1.575	0.195
College diploma	143	27.24	139.36 ± 21.211		
Bachelor	246	46.86	137.50 ± 20.975		
Master or higher	70	13.33	138.56 ± 19.848		
Occupation					
Administrative	16	3.05	146.75 ± 17.613	2.270	0.061
Medical	47	8.95	142.49 ± 20.371		
Clerk	199	37.9	135.96 ± 21.86		
Professional	94	17.9	138.86 ± 19.425		
Freelance and others	169	32.19	140.74 ± 20.315		
Monthly income (yuan)					
< 5000	210	40.00	137.81 ± 22.66	1.680	0.153
5000–	166	31.62	137.71 ± 18.765		
10,000–	87	16.57	140.85 ± 18.385		
15,000–	46	8.76	141.07 ± 20.886		
≥ 20,000	16	3.05	149.69 ± 25.374		
Husband’s educational background					
College diploma below	68	12.95	144.54 ± 18.126	2.269	0.080
College diploma	142	27.05	139.55 ± 20.24		
Bachelor	233	44.38	137.61 ± 22.273		
Master or higher	82	15.62	136.96 ± 18.881		
Husband’s occupation					
Administrative	39	7.43	139.92 ± 20.591	0.431	0.787
Medical	19	3.62	140 ± 25.377		
Clerk	232	44.19	138.5 ± 20.946		
Professional	103	19.62	137.24 ± 17.564		
Freelance and others	132	25.14	140.56 ± 22.345		
Previous breastfeeding experience					
None, first born	307	58.48	131.34 ± 19.282	40.865	< 0.001
Exclusive breastfeeding	139	26.48	150.51 ± 17.904		
Partial breastfeeding	73	13.90	148.56 ± 16.756		
Artificial feeding	6	1.14	142.00 ± 32.472		
Residency					
Registered urban residents	291	55.43	139.62 ± 21.132	0.842	0.400
Registered rural residence	234	44.57	138.08 ± 20.389		
Pregnant women school attendance (times)					
0	415	79.05	136.94 ± 20.805	7.693	< 0.001
1–2	84	16.00	144.56 ± 18.961		
3–4	18	3.43	149.78 ± 19.374		
≥ 5	8	1.52	158.88 ± 15.301		
APP usage (days per week)					
0	56	10.67	134.79 ± 20.532	1.196	0.311
1–2	160	30.48	139.56 ± 21.302		
3–4	104	19.81	141.09 ± 18.905		
≥ 5	205	39.05	138.48 ± 21.348		
Type D personality					
No	342	65.14	142.15 ± 21.025	4.957	< 0.001
Yes	183	34.86	132.91 ± 19.009		

### Breastfeeding competency level of participants

The mean of breastfeeding competency was (138.93 ± 20.798) points, of which "breastfeeding knowledge" dimension was (57.62 ± 9.098), "breastfeeding skill" dimension was (40.71 ± 5.480), "management of breast-milk" dimension was (13.33 ± 3.146), "self-concept and psychology" dimension was (27.27 ± 6.116). This indicating that the breastfeeding competency of pregnant women was at a moderate level (See Table 2).

**Table 2:** Score of each item, factor and total scores of BCS.

Item	Mean ± SD	Rank
1. I know the commencement and termination time of breastfeeding	3.74 ± 0.922	9
2. I could make skin-to-skin contact with my baby while breastfeeding	4.29 ± 0.755	2
3. I know the benefits of breastfeeding	4.38 ± 0.691	1
4. I could be responsive to baby while breastfeeding	4.17 ± 0.817	4
5. I know the merit and demerit of formula as a supplement	3.61 ± 1.009	11
8. I know the precaution and contraindication of drug taking during lactation	3.66 ± 1.013	10
10. I could identify when my baby is hungry	3.50 ± 0.897	12
11. I could identify when my baby is full	3.46 ± 0.887	13
12. I am concerned with baby’s defecation (frequency, nature)	4.04 ± 0.785	5
13. I am concerned with baby’s urine (frequency, color)	4.02 ± 0.803	6
14. I could observe baby’s growth and development on regular basis	4.19 ± 0.713	3
15. I know the commencement time of complementary foods for baby	3.97 ± 0.885	7
16. I know the time when the baby is weaned	3.78 ± 0.889	8
17. I know the way to wean for baby	3.45 ± 0.983	14
43. I know the secretion mechanism of breast-milk	3.35 ± 0.989	15
Factor 1 breastfeeding knowledge	57.62 ± 9.098	**–**
20. I could relieve anxiety and depression during lactation	2.87 ± 0.969	10
26. I am confident to take breastfeeding during night	3.69 ± 0.883	8
34. I am confident to complete breastfeeding	3.94 ± 0.863	6
36. I could proactively acquire of knowledge about breastfeeding	4.11 ± 0.742	4
37. I know the pathway to get knowledge about breastfeeding	3.89 ± 0.843	7
38. I could insist on breastfeeding in public areas	3.32 ± 1.026	9
39. I could get support from my family	4.09 ± 0.776	5
40. I think breastfeeding is my responsibility	4.23 ± 0.797	2
41. I could not temporarily give up breastfeeding for self-image	2.07 ± 1.085	11
42. I think breastfeeding is important	4.32 ± 0.754	1
44. I think breastfeeding is my obligation	4.19 ± 0.845	3
Factor 2 breastfeeding skill	40.71 ± 5.480	**–**
21. I know how to promote breast-milk secretion	3.33 ± 0.919	6
22. I could breastfeed with comfortable positions	3.79 ± 0.812	1
23. I know the way to comfort irritability baby when breastfeeding	3.45 ± 0.91	3
24. I know the way to wake baby up while breastfeeding	3.39 ± 1.588	5
25. I could guide the baby latching on while breastfeeding	3.64 ± 0.914	2
27. I could control the speed of breast-milk secretion	2.98 ± 0.924	8
28. I could correct baby nipple confusion	3.28 ± 0.939	7
29. I know the way to pull nipple out of baby's mouth	3.41 ± 0.995	4
Factor 3 self-concept and psychology	27.27 ± 6.116	**–**
30. I know the way to store breast-milk	3.51 ± 0.932	2
31. I know the way to reheat frozen or refrigerated breast-milk	3.33 ± 0.995	3
32. I could milk extra breast-milk by hand	2.95 ± 1.027	4
33. I know the way to use a breast pump	3.54 ± 0.985	1
Factor 4 management of breast-milk	13.33 ± 3.146	**–**
Total scores of BCS	138.93 ± 20.798	**–**

*SD* standard deviation.

### Univariate analysis of breastfeeding competency level

The dependent variable is the breastfeeding competency score. The independent variables were age, gestational age, educational background, occupation, monthly income (yuan), husband’ s educational background, husband’ s occupation, previous breastfeeding experience, residency, pregnant women school attendance (times), app usage (days per week), and type D personality. Univariate analysis of breastfeeding competency level was carried out. There were statistically significant differences in breastfeeding competency among pregnant women with different characteristics. The results showed an upward trend with increasing age and gestational age. Pregnant women school attendance had a significant impact on the breastfeeding competency of pregnant women. This study showed that pregnant women with type D personality have lower breastfeeding competency than those others (p < 0.05). However, there were no statistically significant differences in pregnant women education and occupation, husband’ s educational background and occupation, and app usage on breastfeeding ability (See Table 1).

### Multivariate analysis of breastfeeding competency level

The score of breastfeeding competency was taken as the dependent variable, and some factors (age, gestational age, previous breastfeeding experience, and pregnant women school attendance) were taken as independent variables for multivariate analysis, after comprehensively considering the clinical practical significance and results of univariate analysis. Multivariate analysis was performed using the enter method to explore the influencing factors of breastfeeding competency level. The results of this study showed that pregnant women with higher breastfeeding competency were found to have a gestational age ≥ 256 days (37 weeks) (B = 8.494, SE = 1.718, p < 0.001); the previous breastfeeding experience were exclusive (B = 17.384, SE = 1.991, p < 0.001) and partial feeding (B = 16.878, SE = 2.391, p < 0.001); participating in pregnant women school 2–3 times (B = 10.968, SE = 4.381, p = 0.013) and ≥ 5 times (B = 13.731, SE = 6.463, p = 0.034). Pregnant women with lower breastfeeding competency were found in women with D-type personality (B = − 6.358, SE = 1.665, p < 0.001). It can explain 25.8% of the variation in the total breastfeeding competency score. There was no multicollinearity in the results, which indicates that all factors in the model were independent predictors of breastfeeding competency (Durbin-Watson value = 1.867, VIF = 1.019–1.262, tolerance = 0.793–0.982). There was no significant interaction between breastfeeding competency and the other factors included in the model (p > 0.05) (Table 3).

**Table 3:** Multivariate analysis of breastfeeding competency level (n = 525).

Factors	Unstandardized coefficients		Standardized coefficients Beta	*T*	*P*	Collinearity statistics	
	B	Std. error				Tolerance	VIF
(Constant)	130.375	1.394		93.545	< 0.001***		
Age (ref. < 35)							
≥ 35	−0.432	2.426	−0.007	−0.178	0.859	0.839	1.191
Gestational week (ref. < 256/36^+6^)							
≥ 256/37	8.494	1.718	0.188	4.944	< 0.001***	0.982	1.019
Previous breastfeeding experience (ref. none, first born)							
Exclusive breastfeeding	17.384	1.991	0.369	8.730	< 0.001***	0.793	1.262
Partial breastfeeding	16.878	2.391	0.281	7.058	< 0.001***	0.894	1.119
Artificial feeding	10.790	7.555	0.055	1.428	0.154	0.949	1.054
Pregnant women school attendance (ref. 0)							
1–2	3.772	2.176	0.067	1.733	0.084	0.961	1.040
2–3	10.968	4.381	0.096	2.503	0.013	0.963	1.039
≥ 5	13.731	6.463	0.081	2.125	0.034	0.976	1.024
Type D personality (ref. No)							
Yes	−6.358	1.665	−0.146	−3.818	< 0.001***	0.971	1.029

*P < 0.05; **P < 0.01; ***P < 0.001.

## Discussion

Pregnant women in third trimester pregnancy had a moderate level of breastfeeding competency. Among the 4 dimensions, breastfeeding knowledge has the highest score, and the management of breast-milk score is the lowest. The results indicate that pregnant women place greater emphasis on breastfeeding knowledge than management of breast-milk, which could negatively impact breastfeeding behavior and lead to a decline in breastfeeding rates. Although pregnant women generally understand the protective effects of breastfeeding and have the high intention of breastfeeding, they are not clear about the detailed knowledge and skills of breastfeeding^26^. When pregnant women have difficulty breastfeeding, they choose to add formula milk to their newborns^27^. The level of breastfeeding knowledge, skills, and intention of supporters also affects the improvement of pregnant women breastfeeding competency^28^.

According to The competency iceberg model, the Conceptual Model of Components of an Enabling Environment for Breastfeeding, and the issues experienced by pregnant women in China^9,13,17,24^, we summarized the factors that include breastfeeding knowledge and skills, self-concept, and traits of pregnant women could influence the breastfeeding competency. So we chose the factors including age, gestational week, educational background, type D personality, and so on of pregnant women or their husbands to investigate the independent risk factors for breastfeeding competency. The analysis showed that higher breastfeeding competency in pregnant women were reported among pregnant women who gestational age ≥ 256 days (37 weeks), the previous breastfeeding experience were exclusive breastfeeding and partial breastfeeding, participating in pregnant women school 2–3 times and ≥ 5 times. Pregnant women with lower breastfeeding competency were found in women who were judged to have type D personality.

This study showed that full-term pregnant women [gestational age ≥ 256 days (37 weeks)] had more breastfeeding competency than pregnant women in nearly full-term [gestational age < 256 days (37 weeks)]. This result may be explained by different attention to different gestational weeks knowledge among pregnant women and health care personnel. In the 10-week prenatal education designed by Leslie McCormack according to Maslow’ s Hierarchy of Needs, breastfeeding is placed in the second half of the whole course because of the development of gestational weeks^29^. Meanwhile, pregnant women have absolute autonomy in choosing prenatal education, and the development of their perinatal health care capacity is more controlled by themselves. Nearly full-term pregnant women pay more attention to fetal growth and development, while full-term pregnant women pay more attention to breastfeeding^30^. Therefore, different level of the breastfeeding competency of pregnant women in different gestational weeks may be because of the changing attention of pregnant women and health care personnel to perinatal knowledge. Pregnant women in nearly full-term delay or give up breastfeeding due to their low breastfeeding competency whose baby may not be protected by breast milk without delay against various risks associated with premature birth.

Experienced pregnant women who breastfed previously who were more likely to start or continue breastfeeding than inexperienced pregnant women^31^, similar to the results of this study. Previous breastfeeding experience could improve pregnant women breastfeeding competency by increasing breastfeeding knowledge, skills, and attitudes, which directly or indirectly affects breastfeeding behaviors^32^. In addition, guiding pregnant women to recall previous positive breastfeeding behaviors can increase breastfeeding self-efficacy^23,31^. Therefore, the importance of breastfeeding behaviors should not only be emphasized in prenatal education to help experienced mothers start breastfeeding. It should be more important to pay attention to breastfeeding behaviors of primiparas to help the development of breastfeeding competency in re-pregnancy and reduce the risk of delaying or giving up breastfeeding.

Prenatal education for pregnant women with breastfeeding as its content could promote the development of their breastfeeding competency. Health education in the healthcare centers was more effective than in other departments in the hospital, which is reflecting the importance of pregnant women school in improving breastfeeding competency and behaviors^33^. The study showed that pregnant women can gain more perinatal knowledge through prenatal education provided by pregnant women school^34^. Breastfeeding courses, support, and information provided by pregnant women school could increase breastfeeding confidence, and provide knowledge and skills to help pregnant women provide breastfeeding for a longer period of time^35^. The research by J L Guo showed that breastfeeding intervention based on the theory of planned behavior can promote breastfeeding by breaking down breastfeeding behavior^36^. Compared with the traditional breastfeeding prenatal education, theory-based education in pregnant women school can increase breastfeeding self-efficacy and improve breastfeeding competency^37^. Therefore, breastfeeding interventions could maximize the effect by using theory-based pregnant women school education.

Type D personality is a special personality type with both negative affectivity and social inhibition^38^. People with type D personality have the feeling of depression, worry, neuroticism, introversion, and often feel unsafe and anxious in interactions with other people^39^. This study showed that pregnant women with type D personality had lower breastfeeding competency than others, which could be explained by the influence of negative psychological on breastfeeding. Pregnant women with an extroverted nature have decisiveness and positive emotions, and show more potential in starting or continuing breastfeeding^40^. In contrast, pregnant women with neuroticism, anxiety, and depression shilly-shally to make breastfeeding decisions and have lower breastfeeding competency than others^41^. The reason may be that pregnant women with an extroverted and openness nature can seek initial common breastfeeding problems more easily and have more sensitive to their children’ s needs than pregnant women with negative psychological, which help them provide early initiation to continued breastfeeding^42^.

The study also had two limitations. First, only pregnant women in the third trimester were invited and two hospitals were included in this study. Although this research content was explained to pregnant women in detail before they were invited to participate in this study, the results is not representative of pregnant women in China and not reflect the influencing factors on breastfeeding competency in the other pregnancy status. So, this limitation may reduce the representativeness of the results in China. Second, the influencing factors included in this study could only explain 25.8% of the variability in the breastfeeding competency score, indicating that other important influencing factors have not yet been discovered. Therefore, more factors such as the quality of the prenatal education provided, the breastfeeding experience of female relatives, pregnant women’ s work should be included to verify the influencing factors of breastfeeding competency.

## Conclusions

Pregnant women in third trimester pregnancy had a moderate level of breastfeeding competency. Gestational week, previous breastfeeding patterns, participation in pregnant women school, and type D personality were independent factors of breastfeeding competency. This provides a basis for health care personnel to improve the breastfeeding rate by adjusting the prenatal education model, improving the level of breast-milk management, and paying attention to type D personality pregnant women and other interventions in future research.At the same time, in order to comprehensively promote the breastfeeding rate from the point of breastfeeding competency, we could control and grasp knowledge, skills, self-efficacy, social support, and other contents together at the same time. Therefore, an intervention study of breastfeeding competency could be considered a new way to improve the breastfeeding rate in the next step. Meanwhile, the influencing factors of breastfeeding competency should be comprehensively considered in the intervention studies to meet the demand for breastfeeding support among different types of pregnant women.

## Data Availability

The data analyzed in this study are not publicly available due to privacy policy, but are available from the corresponding author on reasonable request.

## Abbreviations

BCS: Breastfeeding competency scale
SD: Standard deviations
VIF: Variance inflation factor
SET: Social ecological theory
ANOVA: Analysis of variance
